# The Bells test: assessment of visual-attention disorders in school children (case study from Marrakech, Morocco)

**DOI:** 10.1590/1980-5764-DN-2026-0478

**Published:** 2026-08-24

**Authors:** Hanane Achaq, Ahmed Ahami Omar Touhami, Safia Basla, Gouddar Ghoufrane, Raja Moussaoui, Issam Majid, Omar Amahmid, Youness Rakibi, Zakaria Ouhaz, Said Chakiri

**Affiliations:** 1 Ibn Tofail University, Faculty of Sciences, Biology and Health Laboratory, Morocco.; 2 Ibn Tofail University, Faculty of Sciences, Geosciences and Natural Resources Laboratory, Kenitra, Morocco.; 3 Polydisciplinary Laboratory for Research in Didactic, Education and Training, Department of Life and Earth Sciences, Regional Centre for Education and Training Professions, CRMEF Marrakech-Safi, Ibn Rochd, Marrakech 40000, Morocco.; 4 Sidi Mohamed Ben Abdellah University, Faculty of Technical Sciences, Laboratory for Research in Science and Engineering, Fez, Morocco.; 5 Higher Institute of Nursing and Health Technology Professions, Marrakech, Morocco.

**Keywords:** Students, Visual Attention, Bells Test, Marrakech, Morocco, Estudantes, Atenção visual, Teste de Cancelamento dos Sinos, Marrakech, Marrocos

## Abstract

Various learning disorders affect children without any sensory (vision, hearing), psychiatric, or neurological impairments, even when they have adequate intelligence and a suitable social environment. These children have difficulties in learning to read, write, spell, calculate, express themselves, or concentrate.

**Objective:**

To demonstrate the importance of the Bells test in young students without visuospatial complaints and to evaluate the test in different levels of schooling, age groups, and genders.

**Methods:**

Among the one hundred (100) middle school students enrolled in the second year of middle school, sixty (60) students were selected to be tested using the Bells test. This test consists of a sheet containing 315 small drawings, including 35 bells. The participants are required to circle as many bells as possible (the test time is limited to three minutes).

**Results:**

The percentage of students suspected of having hemineglect is 8.33% or 5/60. Students who forgot their left bells exhibited right hemineglect, while those who forgot their right bells exhibited left hemineglect. Furthermore, 16/60 students surveyed obtained a negative score on the left-right hemineglect calculation, representing 26.66%. Omissions were slightly more frequent on the right side. A statistical analysis revealed a non-significant correlation between the two sexes.

**Conclusion:**

Identifying neuro-visual disorders in school-aged children, regardless of their age, would allow us to prevent these disorders from impacting learning, treat them in a specific manner, and determine the neurological origin of the disorder in a child experiencing difficulties.

## RESUMO.

Diversos transtornos de aprendizagem afetam crianças sem comprometimentos sensoriais (visão, audição), psiquiátricos ou neurológicos, mesmo quando apresentam inteligência adequada e ambiente social favorável. Essas crianças apresentam dificuldades para aprender a ler, escrever, soletrar, calcular, expressar-se ou concentrar-se.

**Objetivo:**

Demonstrar a importância do teste de cancelamento dos sinos em estudantes jovens sem queixas visuoespaciais e avaliar o teste em diferentes níveis de escolaridade, faixas etárias e sexos.

**Métodos:**

Entre os cem (100) estudantes do ensino fundamental matriculados no segundo ano do ensino médio fundamental II, sessenta (60) foram selecionados para serem avaliados por meio do teste de cancelamento dos sinos. Esse teste consiste em uma folha contendo 315 pequenos desenhos, incluindo 35 sinos. Os participantes devem circular o maior número possível de sinos (tempo limitado a três minutos).

**Resultados:**

A porcentagem de estudantes com suspeita de heminegligência foi de 8,33% (5/60). Os estudantes que omitiram sinos do lado esquerdo apresentaram heminegligência direita, enquanto aqueles que omitiram sinos do lado direito apresentaram heminegligência esquerda. Além disso, 16 dos 60 estudantes avaliados obtiveram escore negativo no cálculo de heminegligência esquerda-direita, correspondendo a 26,66%. As omissões foram ligeiramente mais frequentes no lado direito. A análise estatística revelou correlação não significativa entre os sexos.

**Conclusão:**

A identificação de transtornos neurovisuais em crianças em idade escolar, independentemente da faixa etária, possibilitaria prevenir o impacto desses transtornos na aprendizagem, tratá-los de forma específica e determinar a origem neurológica do transtorno em crianças com dificuldades.

## INTRODUCTION

Visual attention, also known as visuo-attention, is fundamental. Irrelevant stimuli are not consciously processed. This attention is the basis for all intentional visual tasks (looking, exploring a visual scene, reading, memorizing, etc.). It initiates the various processes that visual information must undergo^1^. In school-aged children, vision is generally assessed only in terms of visual acuity. Visuo-attentional disorders are therefore poorly investigated, diagnosed, and treated^2^. The study of visuo-attentional disorders in children involves early screening, assessment using specific tests, and appropriate treatment.

These disorders manifest as difficulties in reading, copying, or visually exploring, and can be assessed by orthoptists, psychomotor therapists, or occupational therapists. Several studies^3^ indicate a significant association between vision abnormalities and a higher risk of learning/reading difficulties leading to academic delay^2^. Visuospatial disorder is a symptom whose characteristics are difficult to determine^4^. The present paper also aims to draw attention to the concept of psychomotor skills, which is one of the most recent concepts in the educational and therapeutic spheres.

The psychomotor functioning of an individual is assessed through neurocognitive tests designed to evaluate and screen for potential psychomotor disorders^5^. Early identification of such disorders is crucial, given their significant impact on quality of life - particularly for preschool and school-age children - since they can influence both educational outcomes and overall developmental health. Visual attention is a neurocognitive function that underlies many behaviors. It supports the effective operation of other functions, such as memory and reasoning, which highlights its fundamental importance. Among its roles, it allows individuals to select a stimulus voluntarily and maintain attentional orientation over time^6^. To enable early and specific detection of visual attention disorders in school-age children, we assessed their psychomotor skills and visual attention using the Bells test to identify potential disorders and prevent subsequent difficulties in their schooling and overall health.

## METHODS

### Study setting

This study was conducted in a middle school located in a rural area, north of the city of Marrakech (Morocco), on the right bank of the Tensift River, with a total population of 36,493 inhabitants according to the 2024 population census^7^.

### Population and sample

Sixty (60) learners out of the one hundred (100) enrolled in the second year of middle school, aged 12 to 16, participated in the study. The sample consisted of 21 boys and 39 girls. We selected this group based on the results obtained by the 100 learners evaluated in continuous assessment exams, as well as on our classroom observations during the Life and Earth Sciences (SVT) learning activities. These observations focused on the learners’ perceived control over tasks, as well as the organization and work strategies they adopted.

According to Viau^8^, perception of controllability refers to a learner’s perception of the degree of control they have over the course and consequences of an activity they are assigned. As noted by Tardif^9^, McCombs^10^, and Candy^11^, factors influencing this perception include the learners’ sense of competence and their attributional beliefs. In academic settings, learners propose various explanations for their successes or failures, which can be classified along three dimensions: the locus of the cause, the stability of the cause, and the controllability of the cause. These perceptions are associated with past experiences and consequently shape the future engagement and actions of learners, particularly with respect to their sense of controllability.

In accordance with the research inclusion criteria, all participants in each class aged 12 to 16 years were tested. Furthermore, the selected group did not have any neurological disorder or psychiatric conditions and did not take any medication likely to affect cognitive functioning.

### Bells test

**Figure 1.**
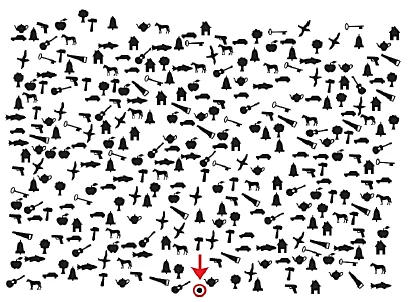
The Bells test sheet.

Source: Adapted from Gauthier et al.^13^

**Figure 2.**
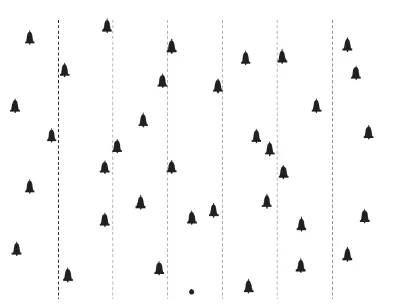
Examiner’s rating sheet.

Source: Adapted from Gauthier et al.^13^

The Bells test was designed to assess attentional abilities and visual hemineglect. It consists of a sheet containing 315 small drawings, including 35 bells. The participant (learner) is asked to circle all the bells on the sheet without wasting time (Figures 1 and 2). Apraxia must be ruled out, as it can affect the validity of the results. The test cannot be completed by a caregiver. Participants must be able to hold a pencil and visually distinguish distracting elements in order to complete the test. The test cannot be used to distinguish between sensory neglect and motor neglect. The examiner then gives the following instructions: “Your task will consist of circling with the pencil all the bells that you will find on the sheet that I will place in front of you without losing time. You will start when I say ‘GO’ and stop when you feel you have circled all the bells. I also ask you to avoid moving or bending your trunk if possible.” If a comprehension problem is present. The examiner has to demonstrate the task. The task sheet is thus given after the instructions. The examiner holds the scoring sheet away from the view of the subject and with the dot towards the subject. This up-side down position facilitates the examiner’s task. After the starting signal, the examiner notes by successive numbering on his sheet (e.g. I, 2, 3...) the order of circling of bells by the subject. If the subject circles another element, the examiner indicates on his scoring sheet by the appropriate number the approximate location. The subsequent bell receives the next number. If the subject stops before all bells are encircled, the examiner gives one and only one incitement in the following terms: “Are you sure that· all bells are now circled’? Verify again.” After the incitement, the order of numbering is still pursued but the numbers are encircled or underlined. The task is completed when the subject stops his activity.

### Statistical analysis

Data analysis was performed using IBM SPSS (Statistics Base 22.0) software and Excel 2010 software for graphical representations. Student’s t-test was used to determine the probability that two data sets were different. p-values less than 0.05 were considered statistically significant.

The variables compared were age, gender, the difference in omissions per active hand, and the difference in omissions over time. The chi-square test was also used to measure the significance of the differences and to compare independent samples. The tables report p<0.001, along with totals and summary statistics.

### Ethical considerations

The study received approval from the Ethics Committee of the Department of Biology, Biology and Health Laboratory, Unit of Neuroscience and Nutrition, Ibn Tofail University, Faculty of Sciences, Kenitra, Morocco. Data confidentiality was ensured by anonymizing the study participants.

## RESULTS

Sixty (60) learners took the Bells test. Table 1 illustrates the distribution of the 60 learners by age and gender.

**Table 1.** Number of individuals in different age groups (The Bells test) and descriptive statistics for age in years.

Age group (years old)	Boys	Girls	Total	p-value*
12-13	00	22	22	<0.001
13-16	21	17	38	<0.001
Total	21	39	60	
**Age in years**				
Number	60			
Average	14.01 years			
Median	14.00			
Mode	13.00			
Standard deviation	0.99			
Minimum	12.00 years			
Maximum	16.00 years			

Note: *p-values lower than 0.05 were considered statistically significant.

Source: The authors.

An omission of six (6) or more bells on the left or right half of the sheet indicates unilateral spatial neglect. In the study population, learners suspected of having hemineglect were 8.33% or 5/60 (Table 2).

**Table 2.** Hemispatial neglect among the learners studied (n=60).

Staff (5 people)	Number of omissions	Types
03	06	Left
01	08	Left
01	12	05 people with right type omissions and 07 people with left type omissions

Source: The authors.

Learners who forgot bells on the left side have right hemispatial neglect, while learners who forgot bells on the right side have left hemispatial neglect. In addition, 16/60 learners obtained a negative score in the calculation of left side omissions-right side omissions (OG-OD), representing 26.66%. Omissions were slightly more significant on the right side. Table 3 illustrates the variation in the average total number of omissions according to the age group.

**Table 3.** Total omissions and difference in omissions by age.

Age group (years old)	Average total omissions per student	Sum of the difference of omissions	p-value*
12-13	2.04	-15	<0.001
13-16	1.97	-5	<0.001

Notes: *p-values lower than 0.05 were considered statistically significant.

Source: The authors.

**Figure 3.**
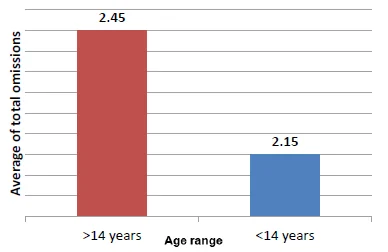
Average total omissions by age group in the study population (n=60).

Source: The authors.

Figure 3 shows that age influences the total number of bell omissions. The number of omissions decreases with age (<14 years=2.45 vs. ≥14 years=2.15). This result can be explained by the students’ relative immaturity, which improves with age.

The results in Table 4 show that the difference in negative OG-OD omissions indicates that omissions are more numerous on the right side in girls than in boys (-03 for boys and -17 for girls).

**Table 4.** Average total omissions and difference in omissions by gender (n=60)

Sex	Average total omissions	Sum of the difference in omissions	p-value
Male	02.28	-03	<0.001
Female	02.07	-17	<0.001
Total	04.35	-11	

Note: *p-values lower than 0.05 were considered statistically significant.

Source: The authors.

**Figure 4.**
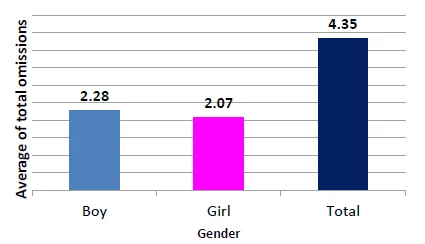
Average number of omissions by gender (n=60).

Source: The authors.

The results shown in Figure 4 indicate that total omissions of bells are more significant among girls than boys. On the other hand, execution time does not appear to differ according to age and gender. The Bells test was able to distinguish between patients with right brain lesions and those with left brain lesions. The difference in negative OG-OD omissions indicates that the mean total omissions are higher in right-handed learners (n=56) compared to left-handed learners (n=4) (Table 5). The Bells test should be used as a screening tool rather than for clinical diagnosis of unilateral spatial neglect.

**Table 5.** Average total omissions and difference in omissions per active hand (n=60).

Active hand	Average total omissions	Sum of the difference in omissions	p-value
Right	02.23	-22	<0.001
Left	01.00	02	<0.001
Total	03.23	20	
**Execution time (min)**	**Average total omissions**	**Sum of the difference in omissions**	**p-value**
≤6	02.09	-15	<0.001
>6	02.22	-05	<0.001
Total	04.31	-20	
**Number of omissions**	**Left-handed omissions (LHO%)**	**Right-hand omissions (RHO%)**	**p-value**
0	06 (10.00)	08 (13.33)	<0.001
1-5	15 (25.00)	26 (43.33)	<0.001
≥6	01 (01.66)	04 (06.66)	<0.001

Note: *p-values lower than 0.05 were considered statistically significant.

Source: The authors.

According to the results in Table 5, the negative OG-OD omission difference indicates that the average total number of omissions is higher among learners who completed the test in ≤6 minutes (n=33) compared to learners who completed the test in >6 minutes (n=27). Table 5 shows that 1.66% (1/22) of subjects have a left unilateral spatial neglect profile, compared to 6.66% who have a right unilateral spatial neglect profile.

## DISCUSSION

This study focused on implementing a screening tool for visual attention disorders in schoolchildren in the Marrakesh region (Morocco). To this end, the Bells test was used to assess the impact of visual attention deficits on learning, particularly reading, which refer to visual perception disorders. These disorders specifically affect the representation of the visual characteristics of letters in words. Several recent studies highlighted that visuospatial attention deficit is a core component of learning difficulties^1^.

Visuospatial attention refers to the number of distinct orthographic units that can be processed simultaneously in a sequence of words. Joint attention is defined as a triadic interaction in which two people coordinate their attention toward an object of mutual interest. “This skill emerges in children around the age of 9 to 12 months”^12^.

The Bells test was developed by Gauthier et al.^13^. This test assesses the visual attention system. It is a simple but highly effective tool. The Bells test enabled us to diagnose hemineglect in 8.33% of the students. Students who missed more than six (6) bells on the right or left may have right or left hemineglect, depending on the location of the missed bells. This could be caused by malfunctioning or maldeveloped neuronal circuits involved in visual attention processes.

Our results showed that the average score was 32.8, compared to an average score of 33.3 found by Gauthier et al.^13^, an average score of 32.94 found by Rousseaux et al.^14^, and an average score of 29.86 found in the ODYDES study. Furthermore, El Azmy et al.^15^ reported a score of 31.79. The results showed that 8.33% of learners have attention problems. This will have a negative impact on their schooling, leading to an increase in the percentage of academic failures. Statistical analysis reveals that the effect of gender on the Bells test scores is insignificant. This is consistent with the findings of studies conducted by Gauthier et al.^13^ and Rousseaux et al.^14^

Gauthier et al.^13^ examined the Bells test in 59 participants: 20 controls, 19 with right-brain lesions, and 20 with left-brain lesions. A statistically significant difference in mean scores was observed between the group with right brain lesions and the group with left brain lesions. Vanier et al.^16^ administered the Bells test and the Albert’s test to 40 adults without neurological disorders and 47 patients who had suffered a stroke in the right hemisphere. They found that 38.3% of patients were diagnosed with unilateral spatial neglect using the Bells test (with a threshold score greater than or equal to 4) compared to only 10.6% with the Albert’s test (using a threshold score greater than or equal to 2).

The results of this study suggested that the Bells test is more likely to identify the presence of neglect in stroke patients than the Albert’s test. Ferber and Karnath^17^ examined the ability of various elimination and line bisection tests to detect neglect in 35 patients with spatial neglect. The Bells test detected a significantly higher percentage of omitted targets than the other tests (Line Bisection Test and three elimination tests: Letter Cancellation Test, Star Cancellation Test, and Line Crossing Test). The Line Bisection Test omitted 40% of patients with spatial neglect. The Letter Cancellation Test and the Bells Test missed only 6% of cases. Out of a sample of 136 students aged 11 to 19 at a middle school in M’rirt (Middle Atlas, Morocco), the scores obtained by the subjects of this study show that 21.32% of learners have attention problems and 5.88% have a profile of right or left unilateral spatial neglect.

This study also highlights a strong correlation between learners’ academic performance and their scores on the bell barrier test^15^. Furthermore, the results obtained from the bell curve test (BC) scores showed no significant difference between the three areas: urban, pre-urban, and rural in the Gharb region (Morocco)^18^. The bell barrier test showed that 100% of learners in the urban area passed the test. In contrast, only 51.06% and 61.29% of learners from the peri-urban and rural areas, respectively, passed this test^18^. According to the study by Mancuso et al.^19^ in Italy, the linear regression model indicated a significant effect of age (β=0.56, t=3.78, p<0.001) but not education (β=-0.06, t=-0.13, p=0.89) on the execution (or completion) time. Based on these results, the researchers developed the following conversion formula:

expected execution time=94.03+0.56×age

Next, the maximum execution time beyond which performance can be considered pathological was calculated using the following formula:

maximum execution time = expected execution time + 1.96 × 48.1 (standard deviation of residuals)

The findings of Mancuso et al.^19^ provide a solid basis for identifying pathological performance using the Bells test. Three predictors (i.e., gender, age, and education) and three dependent variables from the Bells test (i.e., asymmetry score, accuracy score, and execution time) were taken into account. As highlighted in previous studies^13^ ^,^ ^14^ ^,^ ^15^ ^,^ ^16^ ^,^ ^17^ ^,^ ^18^ ^,^ ^19^ ^,^ ^20^ ^,^ ^21^, the asymmetry score, the accuracy score, and the execution time are valid indicators of neglect. In particular, the total accuracy score (i.e., the total number of targets crossed out) measures selective attention, indicating the extent to which the participant can detect targets among distractors. In addition, the asymmetry score (i.e., the difference between omissions in the left and right columns) allows for a more detailed quantification of the difference in target detection. It should be noted that in healthy individuals with entirely symmetrical performance, the asymmetry score cut-off can be used to detect both a deficit in left hemi-space exploration in patients with right-side brain damage (which is more common) and a deficit in right hemi-space exploration in patients with left-side brain damage^22^.

The results of Mancuso et al.^19^ indicate a significant effect of predictor age on accuracy score and execution time variables, and a marginally significant effect of predictor education on accuracy scores. Our study overcomes previous attempts to provide all the necessary conditions for using the Bells test^13^ ^,^ ^14^ ^,^ ^15^ ^,^ ^16^. In the international literature, the Bells test is one of the best-known and most frequently used tests for assessing visual hemineglect because it is simple to administer and allows clinicians to quickly detect asymmetries in visual search. We propose these thresholds to enable more reliable use of the Bells test in assessing unilateral spatial neglect, also known as “hemineglect”. At the same time, it is important to emphasize that neglect is a graded phenomenon and that the use of a single instrument is inevitably prone to false negatives^23^.

Therefore, it is always advisable to use several instruments with different formats, such as line bisection, reading, writing, constructive praxis, and other cancellation tests. Ecologically valid tasks should also be used, such as the behavioral subtests of the Behavioral Inattention Scale^24^. This would allow for the detection of all clinical manifestations of unilateral spatial neglect and minimize the chances of false negative results^21^. One study noted that the Bells test and the Letter Cancellation Test were more likely to detect the presence of neglect than the Line Bisection Test in 35 patients with spatial neglect^25^.

In conclusion, this study, conducted on a population of sixty (60) middle school students, identified 8.33% with hemineglect. These students, who forgot more than six (6) bells on the right or left, may have right or left hemineglect, depending on the location of the forgotten bells. The Bells test also allows us to evaluate the strategy by numbering the bells in order. This gives us an idea of the method or strategy used by the child to complete this task (organized from right to left, left to right, or randomly). It is difficult to study the strategy using the paper version of the Bells test, but it can be studied effectively with its computerized version. Identifying neurovisual disorders in children, regardless of their age, enables prevention of learning impacts, targeted treatment, and determining the neurological cause in children with difficulties^2^.

Thus, to compensate for limitations in the visual system, children develop visual attention skills at different levels of reading activity when learning to read. Ranging from the orientation of visual information intake to the control of visual processing, we can see how these various skills contribute to the development of efficient reading^26^. Although the results of this work seem promising, caution is advised. Certain events experienced by the participant (learner) during this work may have influenced some of the results. However, case studies are relatively rare in Moroccan schools, and this research is part of a new wave promoting qualitative research in neurocognitive sciences.

## Data Availability

The datasets generated and/or analyzed during the current study are available from the corresponding author upon reasonable request.
